# The Impacts of Bio-Based and Synthetic Hydrogels on Soil Hydraulic Properties: A Review

**DOI:** 10.3390/polym14214721

**Published:** 2022-11-04

**Authors:** Toby A. Adjuik, Sue E. Nokes, Michael D. Montross, Ole Wendroth

**Affiliations:** 1 Department of Biosystems and Agricultural Engineering, University of Kentucky, Lexington, KY 40503, USA; 2 Department of Plant & Soil Sciences, University of Kentucky, Lexington, KY 40503, USA

**Keywords:** super absorbent polymers, soil water retention, hydraulic conductivity, natural, literature review, cellulose-based, starch-based, infiltration, evaporation

## Abstract

Soil hydraulic properties are important for the movement and distribution of water in agricultural soils. The ability of plants to easily extract water from soil can be limited by the texture and structure of the soil, and types of soil amendments applied to the soil. Superabsorbent polymers (hydrogels) have been researched as potential soil amendments that could help improve soil hydraulic properties and make water more available to crops, especially in their critical growing stages. However, a lack of a comprehensive literature review on the impacts of hydrogels on soil hydraulic properties makes it difficult to recommend specific types of hydrogels that positively impact soil hydraulic properties. In addition, findings from previous research suggest contrasting effects of hydrogels on soil hydraulic properties. This review surveys the published literature from 2000 to 2020 and: (i) synthesizes the impacts of bio-based and synthetic hydrogels on soil hydraulic properties (i.e., water retention, soil hydraulic conductivity, soil water infiltration, and evaporation); (ii) critically discusses the link between the source of the bio-based and synthetic hydrogels and their impacts as soil amendments; and (iii) identifies potential research directions. Both synthetic and bio-based hydrogels increased water retention in soil compared to unamended soil with decreasing soil water pressure head. The application of bio-based and synthetic hydrogels both decreased saturated hydraulic conductivity, reduced infiltration, and decreased soil evaporation. Hybrid hydrogels (i.e., a blend of bio-based and synthetic backbone materials) may be needed to prolong the benefit of repeated water absorption in soil for the duration of the crop growing season.

## 1. Introduction

### 1.1. Hydrogels

Hydrogels are three dimensional, hydrophilic, polymeric materials with the ability to absorb and release large amounts of water [1]. Hydrogels have attracted attention from researchers from various backgrounds i.e., medicine, food, pharmaceutical and agricultural industries, with the goal to capitalize on hydrogels’ swelling capacities to solve diverse problems. For example, in medicine, hydrogels have been used as scaffolds to provide mechanical protection to tissues where cells are attached or suspended within the gel [2]. In the food industry, hydrogels are used to encapsulate active ingredients such as probiotics, which will be eventually released slowly in the body of the host [3]. Hydrogels have also been used in agriculture as soil amendments to improve soil hydraulic properties [4]. Agriculture, being one of the highest consumers of water, benefits substantially when soil amendments are added to soil to prevent water stress, or for improving soil physical properties. 

There are several ways of classifying hydrogels. Hydrogels have been classified based on their source, synthesis, or crosslinking [5]. In terms of their source, hydrogels have been described as either natural (bio-based) or synthetic. Bio-based hydrogels are hydrogels that are prepared using natural polymers, while synthetic hydrogels are hydrogels synthesized through chemical polymerization of synthetic monomers [6]. Bio-based hydrogels are prepared using polysaccharides such as alginate, chitosan, and dextran [6], while synthetic hydrogels include poly (ethylene glycol) diacrylate, poly (acrylic amide), and poly (vinyl alcohol) [7]. In this review, we use the term “bio-based hydrogels” to represent the class of hydrogels that contain materials of biological origin. 

Several synthesis routes have been applied by different researchers to produce hydrogels. The synthesis route and backbone polymers used generally determine the classification of the final hydrogel as either bio-based or synthetic. A common bio-based polymer for producing hydrogel is alginate. Alginate/alginic acid is a polysaccharide-based biopolymer consisting of α-l-glucuronic acid (G) and β-d-mannuronic acid (M) moieties [8] found as a structural component in marine brown algae (phaeophyceae), as well as a capsular polysaccharide found in soil bacteria [9]. A common route for preparing alginate-based hydrogels is from reacting aqueous alginate solutions with divalent cations (commonly Ca^2+^) by binding with the G units and forming crosslinks with adjacent G blocks of adjacent polymer chains resulting in a hydrogel [8]. Several researchers have improved the properties of alginate-based hydrogels by including other biopolymers as additives [10,11,12]. Another efficient pathway by which bio-based hydrogels can be produced is through free-radical graft polymerization of vinyl monomers onto a biopolymer backbone e.g., carbohydrates and proteins [13]. According to Duquette and Dumont [13], a carbohydrate biopolymer is dissolved with a suitable solvent, heated to 60–80 °C, and an initiator is added, which eventually decomposes to generate free radicals. The free radicals release hydrogen from the functional groups (–COOH, –OH, –NH2) present in the carbohydrate’s polymer chains leading to a crosslinking reaction between polymerized monomers and the functional groups to produce a 3-dimensional network, forming a hydrogel. In the case of a protein, the protein must first undergo hydrolysis to reduce its molecular weight and expose its functional groups prior to hydrogel synthesis [13].

Recently, researchers have turned their attention to synthetic routes that use lignin as a backbone material [14,15,16,17] for synthesizing hydrogels. Lignin hydrogels can be synthesized using an interpenetrating polymer network and polymerization, crosslinking copolymerization, and crosslinking grafted lignin and monomer [14,18]. Lignin hydrogel synthesis through interpenetrating polymer networks can be achieved by free radical polymerization, whereby the phenolic hydroxyl groups of the lignin forms radicals in the presence of an initiator and subsequent reaction with monomers from a different biopolymer to form grafted polymers by radical reaction [14]. Similarly, there are several pathways for synthetizing hydrogels made solely from synthetic polymers such as poly (vinyl alcohol) (PVA), poly (ethylene glycol) (PEG), poly (ethylene oxide) (PEO), poly (2-hydroxyethyl methacrylate) (PHEMA), poly (acrylic acid) (PAA), and poly (acrylamide) (PAAm) [19]. There have been several reviews in the past that adequately reviewed the synthesis routes of synthetic hydrogels [20,21,22], thus this review will not cover the details of synthesis routes of synthetic hydrogels. However, this review will emphasize the different impacts of bio-based and synthetic hydrogels as a soil amendment.

To increase the productivity of agricultural soils, researchers have focused on applying soil conditioners, a term used to describe a subset of soil amendment that affects the physical and/or chemical properties of soil [23]. However, in literature the term soil amendment and soil conditioners have been used interchangeably. Soil amendments have been used in the past to improve soil fertility by increasing the availability of plant nutrients, increasing soil water, decreasing soil drying, maintaining microbiological activity in soil, and increasing nutrient uptake by plants [24]. Garbowski et al. [24] recently reviewed the application of various organic soil amendments such as compost, biochar, sewage sludge, and algae, in agriculture. Hydrogels are a type of soil amendment that have been applied in agriculture to increase water use efficiency [25], reduce nitrate leaching [26], reduce seepage losses in irrigation reservoirs [27], trap water that would have otherwise leached out of the root zone [28], control release agro-chemicals [29], remediate heavy metals from soil [30], used as a soil microbial inoculant [31], and used to improve soil hydraulic properties [32]. There are currently limited review papers that address the impacts of hydrogels as soil amendments on soil hydraulic properties, thus this review provides a timely critical summary of literature to advance the science of hydrogels as agricultural soil amendments.

### 1.2. Swelling Characteristic of Hydrogels

An important feature of hydrogels is their ability to absorb and trap water into their three-dimensional structure. This property is usually referred to as a hydrogel’s swelling capacity. The swelling capacity is one of the most important metrics used to ascertain how well a hydrogel will perform in retaining water in the soil matrix. High swelling capacities allow hydrogels to be applied in situations where liquids are absorbed from an environment or expelled into that environment [33]. According to Isık and Kıs [34], the swelling characteristics of a hydrogel depend on the nature (i.e., ionic content, charge and crosslinking agent) of the polymer used to synthesize the hydrogel and the prevailing environmental conditions (i.e., pH, osmotic potential and temperature of the solution surrounding the hydrogel). Ghobashy [35] argued that the swelling process of a hydrogel is a transition from solid state to a fluid without dissolution and the two interfaces interact to become a “gel”. The change in volume of a hydrogel is driven by water diffusion and the equation that has been used to describe the mechanism of diffusion of water into the polymeric network of hydrogel is Fick’s first law [36]. Fick’s first law for diffusion in one direction is given as:

(1) $$j=-D\frac{\partial C}{\partial Z}$$

where j is the flux per unit area, D is the diffusion coefficient, C is the concentration of solute, Z is the distance over which the change in concentration is measured and $\frac{\partial C}{\partial Z}$ is the concentration gradient along the Z axis.

**Figure 1:**
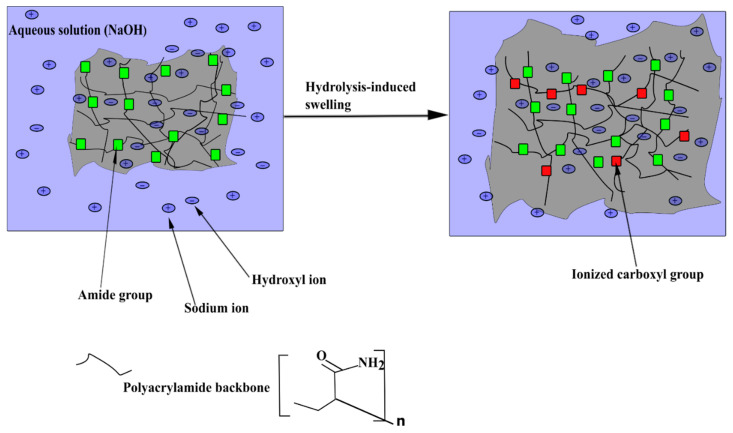
A schematic showing hydrolysis-induced swelling of a polyacrylamide-based hydrogel in the presence of an aqueous solution of NaOH and water molecules; redrawn from Zhou and Jin [37].

Hydrogels need an aqueous environment to swell. According to Zhou and Jin [37], when a polyacrylamide hydrogel is inserted into NaOH with a solvent i.e., water, a hydrolytic reaction takes place whereby the bonds in the polyacrylamide are broken by the hydroxyl ions allowing amide groups from the polyacrylamide chain to react with hydroxyl groups and converted into partially ionized carboxyl groups (Figure 1). The hydrogel then becomes a polyelectrolyte, which allows the hydrogel to absorb considerable amounts of water [37].

Ghobashy et al. [35] have described the swelling process of hydrogels as a continuous swelling and deswelling process affected by the pressure of water molecules on the hydrogel’s 3-dimensional networks resulting in a curve line saw shape (zigzag) swelling curve, known as the hydrogel breath. The swelling capacity of hydrogels is largely dependent on the crosslinking ratio, which is defined as the ratio of the moles of the crosslinking agent to the moles of the repeating hydrogel polymer chains [1]. A small amount of the crosslinker usually results in the formation of water-soluble hydrogels underscoring the need for a critical network density in order to form an insoluble hydrogel capable of swelling with water [38]. On the other hand, excessive crosslinking of hydrogel tends to reduce the swelling capacity of the hydrogels as it reduces the mobility of the polymer chains, thus reducing the swelling capacity [1]. Another factor that affects the degree of swelling in hydrogels are the interactions between the water molecules and the H bonds in the polymer chains of the hydrogel [39]. As water diffuses into the 3-dimensional networks of the hydrogel, primary hydrophilic functional groups such as carboxyl groups (-COOH) and hydroxyl (-OH) present in the polymer chains attach themselves to water molecules through H-bonding [40]. With time, these carboxyl groups begin to hydrate, terminating the H-bonding interactions [35]. Upon the further absorption of water molecules into the 3-dimensional structure, the hydrogel reaches a state where all pores are filled up with water reaching a point known as the equilibrium swelling ratio [41].

Since the swelling mechanism in hydrogels relies on H-bonding between the hydrophilic functional groups and water molecules, the external solution surrounding the hydrogel can also affect the H-bonding process and consequently the equilibrium swelling ratio of the hydrogels [42]. With an increase in the concentration of various metal cations (e.g., free Na^+^ ions, Mg^2+^ and Ca^2+^)), the swelling capacity of a hydrogel is decreased due to the osmotic pressure difference between the polymeric network and the external solutions [43]. The metal cations tend to form metal complexes with the hydroxyl and carboxylic groups present in the hydrogel’s polymer chains [20], thus decreasing the attraction between the hydrophilic functional groups and water molecules [44], which reduces the swelling capacity of a hydrogel.

## 2. Impacts of the Application of Hydrogels on Soil Hydraulic Properties

As a result of the increased attention given to hydrogels and their application in agriculture, several researchers have reported results that show the ability of hydrogels to repeatedly absorb, retain, and release substantial amounts of water relative to the hydrogel’s own weight [45]. The repeated absorption and release cycles imbue in hydrogels the ability to alter movement of water in soil. Soil hydraulic properties refer to the macroscopic interactions between the chemical potential, phase concentration, and the transmission behavior of fluids in soil [46]. Soil hydraulic properties are used to quantify the capacity of a soil to store and transmit water [47]. These hydraulic properties include soil water retention, saturated hydraulic conductivity (*Ks*), unsaturated hydraulic conductivity (*K*), soil water infiltration rate, and soil evaporation rate.

While synthetic hydrogels have been widely researched and claimed to possess superior properties, such as longer durability, high gel strength, and high absorption capacities [48], bio-based hydrogels have also been shown to have high swelling capacities [10,49,50]. The increasing environmental concerns arising from the use of synthetic hydrogels have propelled the research into bio-based hydrogels, since they are presumed to have the advantage of being biocompatible, biodegradable [51] and renewable [52]. Despite the considerable number of studies involved in elucidating the impacts both synthetic and bio-based hydrogels have on soil hydraulic properties, there are limited reviews of their impacts on soil as a function of the hydrogel’s source. In addition, there is a general assumption that since bio-based hydrogels have higher biodegradation rates and extents, then they are inherently better suited as soil amendments than synthetic hydrogels; neglecting the fact that certain bio-based hydrogels, i.e., lignin-based hydrogels, have lower swelling capacities [52], which may need to be improved to have a significant impact as a soil amendment.

Recently, some studies reviewed the effects of hydrogels on water stress management [40] and soil properties (physical, chemical, and biological) [53]. Saha et al. [40] focused on the influence of superabsorbent hydrogels on soil physical properties such as water retention capacity, plant available water, saturated hydraulic conductivity, and soil infiltration. However, the review did not cover an extensive list of relevant studies and included limited discussion of the effects of hydrogel on evaporation. Ostrand et al. [53] summarized the impact of hydrogels on the physical, chemical, and biological properties of soil, but mostly with regards to the depth of application and the rate of application of the hydrogel. This current review focuses specifically on critically summarizing available literature on applications of bio-based and synthetic hydrogels to agricultural soils, with the aim of elucidating if synthetic hydrogels are inherently superior in influencing soil hydraulic properties to bio-based hydrogels, or vice-versa. A discussion of the impacts of both synthetic and bio-based hydrogels on important soil hydraulic properties is needed to better understand the merits and limitations of using synthetic or bio-based hydrogels as soil amendments. First, a thorough review of studies that investigated the impact of synthetic and bio-based hydrogels on important soil hydraulic properties is given. Secondly, a conceptual framework summarizing how hydrogels impact soil hydraulic properties is discussed. The final part of this review identifies research gaps and outstanding questions that need to be answered to move this research area forward. This review does not discuss the impact of hydrogel application to plant growth parameters. The scope of this review also does not include the use of hydrogels as materials for the slow release of nutrients in soil. 

### 2.1. Impact of Hydrogels on Soil Water Retention

**Figure 2:**
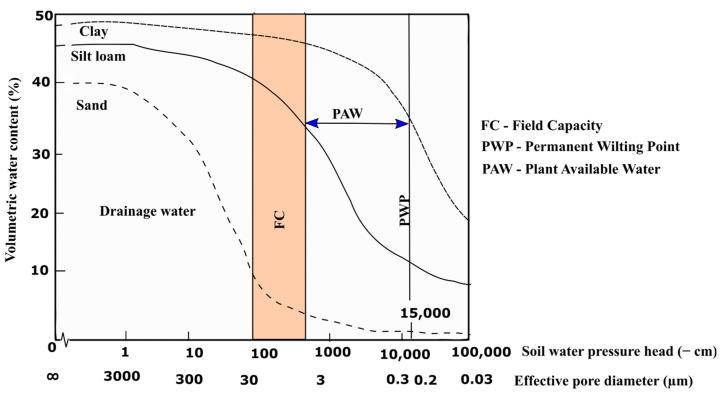
Soil water retention curve showing the relationship between soil volumetric water content and soil water pressure head in sand, silt loam and clay soil (redrawn from Ehlers and Goss [56]).

Soil water retention is the most reported physical property reported in hydrogel literature (summarized in Table 1). Soil water retention refers to the quantity of water a particular soil can hold under given pressure head conditions. Soil water retention is often described as the soil water retention curve (SWRC). Researchers are interested in how the application of hydrogel affects the SWRC (Figure 2) as the results have important agronomic implications. The SWRC describes the relationship between volumetric water content and soil water pressure head at a given location in soil [47,54]. This curve differs for every soil type. Due to capillary forces in soil pores and the adsorption of water on solid surfaces, soil water pressure head in soil is typically negative [47]. As soil water pressure head increases closer to zero, water is mostly held by capillary forces and as soil water pressure head decreases (becomes increasingly negative), water is increasingly tightly bound in the smallest pores in soil, making it difficult for plants to extract.

The part of the SWRC that is most relevant for agricultural decision-making is between the field capacity (FC) and the permanent wilting point (PWP), known as the plant available water capacity (PAWC). The FC of a soil is often described as the amount of water retained by a soil after a rain or irrigation event once drainage has become negligible. The permanent wilting point is the soil water content below which plants wilt permanently [55]. The field capacity of soil will differ according to soil texture. Coarse soil, such as sand, will have a lower field capacity than finer soils like clay. The soil water content available to plant roots (PAWC) is thus defined as the difference in field capacity (FC) minus the permanent wilting point (PWP). From Figure 2, the soil water pressure head can be represented with an effective pore diameter. Thus, as soil water pressure head decreases, soil water is held by smaller pores. When soil is at FC, water in the soil would be held by pores with average diameters corresponding less than 30 µm [56]. As the soil dries and the pressure in the soil approaches the permanent wilting point, water in soil will be held by pores with average diameters corresponding to 0.2 µm [56]. Studies quantifying the effect of hydrogel on soil water retention have predominantly reported significant increases (quantified below) in water retention when the hydrogel application was in sandy soils with hydrogel concentrations ranging from 0.1 to 2% (w/w) [28,57,58,59,60,61,62].

#### 2.1.1. Impact of Bio-Based Hydrogels on Soil Water Retention

From our review, 10 studies applied bio-based hydrogels to different soils. Six of these studies [28,50,59,63,64,65] applied cellulose-based hydrogels to mostly sandy soils and soil water retention increased by a range of 6–500% in the range of soil water pressure head from saturation to permanent wilting point with a concentration of hydrogels between 0.1 and 1.5% w/w. For all the bio-based hydrogels, soil water retention at a given pressure head increased. However, the increased water retention found by Hu et al. [66] was attributed to the added polyacrylamide in the hydrogel, which indicated that bio-based hydrogels can be enhanced by blending them with synthetic hydrogels. The application rate for the bio-based hydrogels ranged from 0.1 to 1.5% (w/w). While most of the studies in this review were conducted in lab conditions, among the bio-based hydrogel studies, Narjary et al. [65] conducted a field study and reported an increase of 6–8% in relative available water capacity of a sandy loam soil with a cellulose-based hydrogel concentration of 5 kg ha^−1^, which was among the lowest increase in soil water retention. However, the hydrogel application rate was very low, with an estimated soil density of 1430 kg m^−3^, 5 kg ha^−1^ would translate to an application rate of 0.0005% (w/w) in the top 7 cm of soil. An earlier study by the same author [64] reported the highest increase of 400% in moisture content in sandy soil in a lab study with a hydrogel concentration of 0.7% (w/w). These results reflect a disparity between lab tests and field tests of hydrogels where conditions are not controlled, and real-world settings may reduce the efficacy of hydrogels.

Sandy soils were used to test the hydrogels in 70% of the studies. There is a dearth of studies examining the impact of bio-based hydrogels on water retention in other soil textures. In addition, most bio-based studies use cellulose-based hydrogels, which have been shown to work effectively at increasing water retention in soil but biodegrade within a few days to a few months [67]. The limited studies using other bio-based materials calls for a shift in attention to the less explored bio-based materials like lignin. For instance, only two studies were found from this review that applied a lignin-based hydrogel [10,38]. Passauer et al. [38] reported a significant increase in soil water content in sandy soils specifically for soil water pressure range between −3 cm and −15,000 cm. At a hydrogel concentration of 0.5% (w/w), which was the highest concentration used, soil water content increased by 14.2% (w/w) at −300 cm. Song et al. [10] applied a lignin-based sodium alginate hydrogel and reported an increase of soil water content by 2.98–8.96% at soil water pressure heads of −1000 cm to −15,000 cm. This was similar to the range of soil water pressure head Passauer et al. [38] observed a 14.2% increase in soil water retention. One reason for fewer studies using lignin hydrogels could be due to lignin’s hydrophobic nature, and its complex and heterogeneous structure, which makes utilization difficult [15]. However, the presence of numerous hydrophilic functional groups (hydroxyl and carboxyl) on lignin’s backbone [52] makes it a suitable candidate for synthesizing hydrogels that could assist in retaining water in soil. The advantage of using lignin is that it can be crosslinked with other materials like sodium alginate to obtain a hydrogel which is biodegradable, non-toxic with high water retention [10] and is a biological waste with minimal alternative uses, unlike starch.

#### 2.1.2. Impact of Synthetic-Based Hydrogels on Soil Water Retention

Synthetic hydrogels, which are mostly made of polyacrylamide or polyacrylate, remain the most widely researched form of hydrogels [68]. A total of 31 studies applied synthetic hydrogels to test their ability to increase soil water retention (Table 1). Thirteen studies applied hydrogels originating from acrylamide/polyacrylamide [45,57,60,69,70,71,72,73,74,75,76,77,78] to mostly sandy soils and sandy loam soils, to quantify their soil water retention ability at an application range of 0–1.5% (w/w). The increase in soil water retention in the soils amended with acrylamide/polyacrylamide-based hydrogel studies ranged from 0.76–330%. Another six studies [70,79,80,81,82,83] applied polyacrylate-based hydrogels mostly to sandy soils at application rates ranging from 0–1% (w/w), which led to a soil water retention increase of 6.2–319%.

The effects of hydrogel on soil water retention seem to be more consistent than for other soil hydraulic properties. However, the impacts have often been significant only for coarse soils i.e., sandy soils. Studies by [28,57,58,59,60,61,62] applied hydrogels at varying concentrations ranging from 0–2.5% (w/w) to sandy soils. Montesano et al. [59] reported a 400% increase in soil moisture at FC with 2% (w/w) application rate while in Banedjschafie and Durner [58], the highest water content was observed at an application rate of 1% (w/w). Studies by Bhardwaj et al. [60] and Andry et al. [28] also showed a significant soil water retention increases after hydrogel treatment to sandy soils.

#### 2.1.3. Impact of Hydrogels on Plant Available Water Capacity (PAWC)

Studies on the impact of hydrogels on PAWC have been consistent i.e., PAWC increases with increasing application rate of hydrogels but to a larger extent in coarse-textured soils [64]. In general, sandy soils exhibit the lowest PAWC [54], hence applying hydrogels to sandy soils may result in higher benefits than in other soils. With hydrogel application rates of 0, 0.1, 0.2, and 0.4% (w/w), Saha et al. [72] observed an increase in PAWC in sandy soil by a factor of up to 3.3 compared to a control treatment. Their study also found that PWP of the sandy soil was delayed by 32 days at the 0.4% hydrogel treatment compared to the control treatment. Their study recommended a 0.1% (w/w) application rate for coarse textured soils and a 0.2% for fine-textured soils, however they only tested a range from 0–0.4% w/w hydrogel. When soils are saturated, hydrogels in the soil absorb a substantial portion of the water while acting as additional pores for storage of the water [84]. As the soil dries, the stored water is released back into the soil for plant roots [40]. Several reasons can be attributed to the ability of hydrogels to retain and release water in the soil matrix. According to Yang et al. [85], the increase in soil water retention with hydrogel application could be due to the strong adsorption and complexing capacities from hydrophilic functional groups, such as hydroxyl, carboxyl, amide, and sulfonic groups from the cross-linking in synthetic hydrogels. Higher soil water retention could also be due to a decrease in median pore diameter with the application of hydrogel [64]. Narjary et al. [64] explains that as pore diameters decrease, smaller retention pores are likely to be found in soil and these pores can hold more water tightly, due to the increase in porosity.

### 2.2. Impact of Hydrogels on Saturated Hydraulic Conductivity

Saturated hydraulic conductivity describes the ability of soil to transmit water when all pores are filled with water [86]. Besides increasing water availability in soils, hydrogels have been shown to affect hydraulic conductivity in soil. The effect of hydrogels on saturated hydraulic conductivity (*Ks*) has been inconsistent though most studies on soil hydraulic conductivity have focused on *Ks* (as opposed to unsaturated hydraulic conductivity). Out of the 14 studies surveyed that investigated the effects of hydrogels on *Ks*, nine of the 14 studies indicated a decrease in *Ks* [10,32,45,57,64,65,74,83,87], however, three studies [28,66,88] reported an increase and three studies reported an initial decrease in *Ks* then a subsequent increase in *Ks* with time [60,88,89]. In two of the studies [60,89], the subsequent increase in *Ks* seem to increase over the original *Ks*. A subsequent increase in *Ks* after an initial decrease could be attributed to the gradual deterioration of the internal molecular structure of the hydrogel caused by the repeated absorption and release of water [89]. The loss of stored water in the hydrogel with time increases percolating water which increase *Ks*.

#### 2.2.1. Impact of Bio-Based Hydrogels on Saturated Hydraulic Conductivity

Of the nine studies that report a decrease in saturated hydraulic conductivity, three of them used bio-based hydrogels [10,64,65]. Narjary et al. [64] applied a cellulose-based polyacrylate hydrogel at 0, 0.5, and 0.7% (w/w) to different soils in a laboratory PVC column experiment. They reported a 55% decrease of *Ks* in the sandy soil with the 0.7% (w/w) hydrogel treatment. In a follow-up field study [65], the authors observed a decrease of 45% and 60% in hydraulic conductivity in a sandy loam with 2.5 and 5 kg ha^−1^ application of a cellulose-based hydrogel, respectively, and reported a 45–60% decrease in *Ks*. Song et al. [10] applied a lignin-based sodium alginate hydrogel to a sandy-loam soil and observed a decrease of 63.2–89.5% in *Ks* of a sandy loam soil with an increase in concentration of the hydrogel from 0 to 0.975% (w/w). The hypothesis for the decrease in *Ks* is that, due to the high swelling rates of the bio-based hydrogels, the hydrogel’s expansion in the presence of water reduced the size of drainage pores [4]. 

It is worth noting that, despite the evidence for a decrease in *Ks* when bio-based hydrogels are amended to soil, two studies [28,66] offered contradictory results. Andry et al. [28] examined the effects of two hydrophilic polymers (carboxymethylcellulose and isopropyl acrylamide) on the *Ks* of a sandy soil as affected by temperature and water quality in a temperature–controlled environment. Their results suggested that *Ks* decreased with an increase in concentration of the two hydrogels. However, they also reported an increase in *Ks* only when soil temperature was increased to 35 °C. The increase in *Ks* as soil temperature increases could be explained by a decrease in soil water viscosity [90]. Similarly, Hu et al. [66] reported a significant increase (91–122%) in *Ks* with the application of bio-based hydrogels at 4 ton/ha to sandy loam soil. They explain their results by hypothesizing that the hydrogel improved soil structure, decreased bulk density and increased porosity which increased *Ks*. 

While the majority of the studies surveyed suggest that bio-based hydrogels reduce *Ks*, the reduction could be hydrogel dependent. Hence, the specific properties of the hydrogel like the swelling capacity in aqueous solutions and in soil could be the major factors that impact *Ks*. A bio-based hydrogel with a high swelling capacity is likely to decrease *Ks* at the moment water first starts to infiltrate soil as more water is stored in the hydrogels and less water is percolated from one soil layer to another. However, when the hydrogels attain their maximum swelling capacities, the stored water is expected to be releases further down the soil layers thus increasing *Ks*. Comparisons of different types of bio-based hydrogels with different properties (swelling capacities) used under similar conditions (e.g., temperature) will help determine the specific factors influencing *Ks*.

#### 2.2.2. Impact of Synthetic-Based Hydrogels on Saturated Hydraulic Conductivity

Much of the evidence for decreases in *Ks* was found in studies that used synthetic hydrogels. A total of six studies [32,45,57,74,83,87] using various synthetic hydrogels definitively argue that hydrogels decrease *Ks*. Four of the six studies applied hydrogels to sandy soils underscoring the need for more studies using other soil textures. Mohawesh and Durner [32] observed a significant decrease in *Ks* by a factor of 3 when a synthetic hydrogel was applied to sandy soil. In a study by Shahid et al. [74], *Ks* was reduced by 16%, 36%, 48%, and 53% for hydrogel application rates of 0.1, 0.2, 0.3, and 0.4%, respectively, using a poly (Acrylamide-co-acrylic acid) based hydrogel. Zhuang et al. [83] reported a decrease in *Ks* by 42.53, 55.45, 87.55, and 96.5% when sodium polyacrylate hydrogel was applied at 0.08, 0.2, 0.5, and 1% (w/w), respectively. The author explained that, as hydrogel concentration increased, the swelling of the hydrogel decreased the paths available for downward movement of water. Smagin et al. [87] noticed that partially swollen hydrogels decreased *Ks* by up to 3.2 times compared to when dried hydrogel was applied, which gave a 1.4-fold reduction in *Ks*. They therefore advised that hydrogels be applied in swollen form to gain full benefits of reduction in *Ks*. Their recommendation is similar to Wei and Durian [91], who emphasized that applying hydrogels in a wet state allowed hydrogels to quickly clump together forming reservoirs in sandy soil pores decreasing the downward percolation of soil water. 

Abdallah [57] applied a polyacrylamide-based hydrogel to a sandy soil at 0 and 0.3% (w/w). Their study showed that *Ks* was significantly reduced, and the reduction was dependent on the particle size of the hydrogel. There was a greater (68.8%) reduction when the hydrogel particle sizes were between 0.8–1.0 mm, compared to hydrogels with particle sizes between 2–4 mm (38.9%). Their result implied that hydrogels with smaller particle sizes may be more useful at reducing *Ks* in sandy soils. It is worth investigating the impact of particle size of bio-based hydrogels to understand how particle size impacts *Ks*, since to the best of our knowledge no study investigates this topic. For the studies surveyed in this review, the particle size distribution of the hydrogels was rarely reported and so there is a question as to the link that particle size distribution of bio-based hydrogel has on the impact on soil hydraulic properties. 

Other studies using synthetic hydrogels have also reported a decrease in *Ks,* but the decrease was not consistent with all application rates neither was it true for the entire duration of the study. Han et al. [89] investigated the effect of different synthetic hydrogel types (Acrylate Sodium Co-polymers (ASC) and Polyacrylamides (PAM)) on *Ks*. Their results suggest that *Ks* decreased sharply on initial hydrogel application, but *Ks* then gradually increased with time. Initially, the swelling of hydrogels led to the blockage of soil pores as the hydrogel attains its maximum swelling capacity, the eventual release of stored water increased *Ks.* In addition, repeated absorption and desorption of the hydrogel resulted in a loss of swelling capacity in the hydrogel thus soil pores that were previously occupied by the swollen volume of the hydrogel were unblocked, and *Ks* increased. 

#### 2.2.3. How Does Application of Hydrogel Affect Saturated Hydraulic Conductivity?

The pressure exerted above the location of a hydrogel can influence *Ks*. Bhardwaj et al. [60] reports an initial decrease of *Ks* with a subsequent increase due to pressure from the soil above the hydrogel causing it to drain water. Hussein et al. [88] showed a decrease in *Ks* (53.68–87.19%) at low concentration of the hydrogel (0.5 and 1% (w/w)) and an increase (107.6–516.3%) at higher concentration (2% (w/w)). The authors attribute the decrease in *Ks* to a reduction in the pore spaces between the soil particles and aggregates caused by swelling in the hydrogel, which blocks movement of water. The authors argue that the higher concentration led to weaker hydrogel soil matrix, which was unable to withstand the hydraulic head exerted by the soil above. It could be argued that the influence of synthetic hydrogels on *Ks* is directly dependent on the residence time of the hydrogel in soil as well as the concentration. The longer the hydrogel stays in the soil, the lower its efficacy at reducing *Ks*. Secondly, as you increase the concentration of the hydrogel, *Ks* reduces up to a certain threshold concentration at which point *Ks* starts to increase drastically.

A possible explanation for the contradictory results regarding the effect of hydrogel on *Ks* could be that due to restricted swelling caused by the pressure exerted from the soil layers above the hydrogel in the soil [40]. For instance, when hydrogel is placed at a depth below the surface of the soil, it begins to swell by absorbing water into its 3D network. To keep the water absorbed in the hydrogel and at that specific depth, the weight of the hydrogel must withstand the weight of soil exerting the downward pressure on the hydrogel. However, with time, the weight of the hydrogel decreases as water gradually moves out of the hydrogel into the surrounding soil due to an increase in matric suction in the soil. At this stage, the ability of the hydrogel to hold onto water now depends on the load applied by the upper layer [92,93] coupled with the matric suction in the soil due to soil drying. These two forces eventually overwhelm the strength of the hydrogel causing the water to drain out creating additional pores through which percolating water drains, thereby increasing the hydraulic conductivity as a result [40].

The hydraulic conductivity decreases considerably as soil becomes unsaturated since less pore space is filled with water, the flow paths become increasingly tortuous, and drag forces between the fluid and the solid phases increase.

### 2.3. Impact of Hydrogel Application on Unsaturated Hydraulic Conductivity

Unlike *Ks*, unsaturated water flow is a process that occurs when the water phase is bound partially by soil particles and partially by an interface with the air phase [94]. Unsaturated hydraulic conductivity generally decreases drastically as soil dries and increasingly fewer pores are filled with water. The decrease in *K* is generally attributed to only small soil pores contributing to water flow which increases tortuosity and the drag forces between water and the soil particles [47]. Like *Ks*, unsaturated hydraulic conductivity (*K*) is important for the movement of water in soil and more than *Ks* in field conditions [86], although fewer studies have investigated the impacts of hydrogels on *K*. A survey of literature found two studies since the year 2000 that measured *K* after applying hydrogel [71,87]. Both studies reported a decrease in *K* with the application of hydrogel. Liao et al. [71] calculated *K* from unsaturated diffusivity measurements of a sandy loam soil when a synthetic polyacrylamide and acrylic acid-based hydrogel were applied at rates of 0, 0.01, 0.03, and 0.06% (w/w). The *K* values were then plotted against the volumetric water content of the samples measured from a range of days from 0 to 120. Their results reveal a decrease in *K* of 85.5 to 94.1% on day 0, 75.1 to 82.9% on day 30 and 65 to 76.2% on day 50. Smagin et al. [87] suggested that at high matric potentials i.e., >−10 to −15 kPa, *K* was reduced up to 2–3 times at hydrogel concentrations ranging from 0.01–0.05% (w/w) and a reduction of 10–50 times at 0.1–0.2% hydrogel concentration. However, at low matric potentials i.e., −20 to −700 kPa, *K* increased with an increase in hydrogel application rate. 

In general, when soil is saturated or near saturation, there are an abundance of conducting pores for water to move through soil thus an increase in hydraulic conductivity is observed. Eventually, as conditions around the soil become unsaturated and tortuous, a decrease in *K* is observed. A hydrogel which can retain bound water for a period of time could gradually release the bound water during extremely dry conditions, which creates a wider path/increase cross sectional area for the movement of water. Field soils where hydrogels may be applied will mostly be limited by water and constantly be in an unsaturated state hence the importance of more studies investigating effect of hydrogel application to unsaturated hydraulic conductivity.

### 2.4. Impact of Hydrogels on Soil Water Infiltration

Infiltration refers to the entry of water into soil and subsequent downward movement [95,96]. Soil water content, suction head, temperature, rainfall intensity, and soil texture all influence the soil infiltration rate [97]. For example, coarse textured soils have large pores, which allow water to quickly move below the reach of crop roots. A review of literature corroborates a decrease in infiltration rate with an increase in hydrogel application rate [73,83,98,99,100]. Studies have mostly agreed that the swelling process of hydrogels reduces the cross-sectional area of larger pores in soil serving as a barrier to the downward movement of water. 

From the eight studies surveyed for this paper shown in Table 1, only one study investigated the impact of a bio-based hydrogel (Poly-γ-glutamic acid-based hydrogel) on the infiltration rate of sandy loam soil [98]. The remaining seven studies all used either polyacrylamide, polyacrylate, or acrylic acid derived hydrogels to apply to soil to study their impacts on the infiltration rate of mostly sandy soils. With application rates of 0, 0.08, 0.2, 0.5, and 1% (w/w), Zhuang et al. [83] observed a decrease in the migration velocity of water into the deep soil layers, while also decreasing the infiltration rate in sandy soil. Three studies [98,99,100] applied hydrogels at rates ranging from 0–1.17% (w/w) to mostly loam soils. Guo et al. [98] concluded that hydrogels decreased the infiltration volume of water, thereby increasing soil water at field capacity. Lentz [99] emphasized that, initially, the added hydrogel may decrease the seepage rate of water by absorbing water and preventing downward percolation, however, in the long term, it is the change in pore-size distribution of soil by hydrogel amendment that will reduce infiltration. Reddy et al. [100] compared the infiltration rate of sandy loam soil amended with four different hydrogels. Their study reports a decrease in infiltration rate of 90% in the best performing hydrogel. Hydrogel reduces the infiltration rate by altering the pore structure [40,99] of soils, especially in sandy soils, where bigger drainage pores are reduced to smaller retention pores.

### 2.5. Impact of Hydrogels on Soil Water Evaporation

Evaporation is a process that occurs when liquid water changes into water vapor and diffuses into the atmosphere [101]. There are three stages of soil evaporation. Stage 1 is where soil is sufficiently wet, so water is readily available at the surface for evaporation [102]. There is a high evaporation rate in stage one, that is determined by the water vapor saturation deficit of the air above the soil. One reason for the high rate of evaporation during stage one is that the soil is saturated, and evaporation begins at the surface of the soil, caused by environmental factors such as atmospheric temperature, wind speed, and humidity [103]. During stage 2, evaporation shifts from the surface water to the sub-surface water, resulting in the formation of a dry surface layer [102]. The soil starts to heat up and the water in the soil profile is unable to move to the surface of the soil fast enough to meet the demands of the evaporation at the surface [103]. Finally, during stage 3, water is transported through the soil as water vapor and follows molecular diffusion within the soil [103]. The rate of water moved is very low at this stage. 

Most studies contend that hydrogels retain water and reduce evaporation (Table 1). From our review, one study [98] investigated the effects of a bio-based hydrogel (Poly-γ-glutamic acid-based hydrogel) on evaporation and was the only study that argued that hydrogels increased the evaporation rate. Guo et al. [98] tested a poly-γ-glutamic acid-based hydrogel on soil evaporation by filling small round PVC columns with hydrogel-soil mixtures at rates of 0, 0.05, 0.10, 0.15, and 0.20%. The experiment occurred in a constant temperature incubator at 50 °C. Evaporation was then measured by the change in mass of the samples every 12 h. Their results indicate that the poly-γ-glutamic acid-based hydrogel increased cumulative evaporation in soil compared to a control treatment. The authors attributed the increase in evaporation to the increase in water storage in the soil because of the hydrogel, which provides water for evaporation to easily occur. In addition, as water vaporizes, the hydrogels lose water and shrink, which may increase soil porosity and the contact area between air and soil particles, thus increasing evaporation [70,98].

On the other hand, the remaining six studies that investigated the effects of hydrogels on soil evaporation [45,62,104,105,106,107] indicated a decrease in soil evaporation with the application of different synthetic hydrogels. In a laboratory experiment, Yang et al. [105] filled a rectangular box with sand to a height of 117 cm. A 10 cm soil-hydrogel layer was placed 20 cm below the soil surface and the rectangular sand box was irrigated. After 4 and 9 days of evaporation, the water content was highest in the soil-hydrogel layer, followed by the bottom layer, and the surface layer had the lowest water content. In a similar study by Zhao et al. [106], a soil-hydrogel layer of 10 cm was placed at a depth of 10 cm with 10 cm sand above and 40 cm of sandy loam soil below. Application rates of 0.2, 0.5 and 1% (w/w) significantly decreased evaporative loss with an increased water storage at the 2 and 18 cm depths after 10, 20, and 30 days of evaporation. Two other studies [104,108] also confirmed the ability of hydrogel-soil mixture to retain more water after drying in an oven at 60 °C for 5 h. Yu et al. [104] suggested that after applying acrylamide-based hydrogel at a rate of 5 g hydrogel/kg soil, the amount of retained water in the soil increased thus extending the first stage of evaporation.

Hydrogels may alter the drying stages of soil by increasing water storage. By placing hydrogels at a specific depth near the surface of the soil, hydrogels can reduce hydraulic conductivity [105], thus keeping more water in the topsoil for a longer time. This prolongs stage 1 drying since there would still be enough water at the surface of the soil. Secondly, as the soil profile gradually dries under natural conditions and enters stage 2, hydrogels can intercept the movement of water upwards as some water will be absorbed and kept at the level just beneath the soil surface. The extent of the changes in soil evaporation will, however, depend on the type and amount of the hydrogel applied.

### 2.6. Summary of How Hydrogels Influence Soil Hydraulic Properties

**Figure 3:**
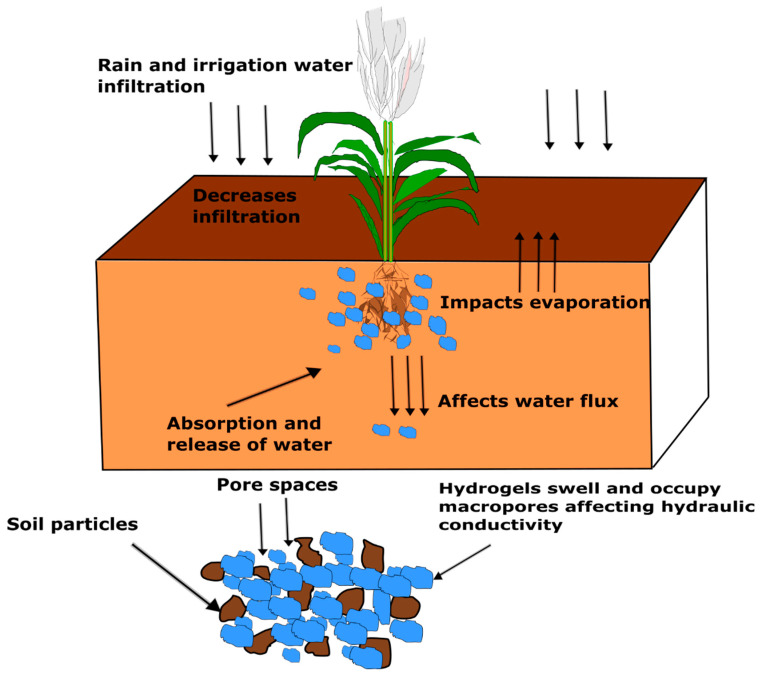
Conceptual diagram describing the impact of hydrogel on different soil hydraulic parameters, (redrawn and modified from Saha et al. [40]).

Figure 3 conceptually illustrates how hydrogels may impact the reviewed soil hydraulic properties. When rain falls or soil is irrigated, infiltration is initially high and water percolates into the soil profile making it available to plant roots until the soil becomes saturated, and infiltration stops. Near the roots of the plant, hydrogels swell by absorbing water. As the soil becomes unsaturated and soil water pressure head decreases, the water absorbed by the hydrogel is slowly released into the soil matrix making it available for plant roots to use. Some water also leaves the soil into the atmosphere through evaporation. Within large soil pores, the swollen hydrogel could prevent the downward flux of water thus decreasing *Ks*. However, the soil water pressure head eventually decreases, and soil pores gradually become air filled, and the flow path of water becomes tortuous as drag forces between the water and soil phase increases [47]. Assuming hydrogels can retain water in saturated conditions and release that water when soil water pressure head decreases, then it is expected that the gradual release of water from the hydrogel creates a less tortuous path for water flow hence potentially increasing unsaturated hydraulic conductivity.

A noteworthy trend from this review is that, as soil water retention increased because of the application of both bio-based and synthetic hydrogels, *Ks* decreased. This trend was noticed in 83% of the studies that measured the effects of hydrogel on both soil water retention and *Ks*. The increase in soil water retention ranged from 0.76–318.89%, while the decrease in *Ks* ranged from 9–708%. This trend implies that as the hydrogel swells and holds water at the position of the soil profile where it is placed, the movement of water is limited as the swollen hydrogels occupy the drainage pores, thus decreasing *Ks*. Another trend worth mentioning is that as soil water retention increased with application of hydrogel, cumulative evaporation decreased in three out of four studies that quantified both soil water retention and cumulative evaporation. However, this trend may only be valid if the hydrogel is positioned strategically at a location below the surface of the soil. When placed near the soil surface, soil water retention increased accompanied by increased cumulative evaporation [98].

## 3. Important Factors to Consider When Applying Hydrogels to Influence Soil Hydraulic Properties

(i) The swelling capacity of a hydrogel in aqueous solutions and in soil is an important indicator of performance. The swelling capacity of a hydrogel enables the hydrogel to absorb and expel water from its environment [33]. From this review, the swelling capacity of a hydrogel directly affects all the soil hydraulic properties discussed. Since hydrogels will have to be in the presence of soil to influence soil hydraulic properties, it is worth quantifying the swelling capacity of the hydrogel when confined in soil. A hydrogel with a higher swelling capacity will absorb more water in soil which increases water retained in the soil. The increase in surface area of the hydrogel with swelling also impedes the downward movement of water, thus decreasing hydraulic conductivity and soil water infiltration. Higher swelling in hydrogels also leads to higher water storage, which reduces evaporation when hydrogels are placed at an appropriate depth in soil.
(ii) Swelling characteristics when confined under soil pressure impacts hydraulic properties. An ideal hydrogel should be able to withstand the pressure exerted by the surrounding soil. Hydrogels should be designed to be able to absorb water causing it to swell, changing the shape, mass, and volume of the hydrogel in the process, even at depth within the soil. According to Misiewicz et al. (2020), during the swelling of hydrogels, the hydrogel-soil mixture exerts pressure on the top layer of the soil. Due to this pressure exerted by the hydrogel during swelling, the hydrogel can repeatedly absorb and release water in soil against the pressure exerted by the soil. Misiewicz et al. (2020) further explains that the cause of the pressure exerted by the hydrogel during swelling depends on the available soil pore volume and the grain size distribution of the hydrogel. Similarly, Louf et al. [117] demonstrated that in a three-dimensional granular medium, e.g., soil, the extent of swelling in a hydrogel depends on the antagonistic competition between the force exerted by the hydrogel osmotic pressure and the force exerted by the surrounding soil. While these studies [93,117,118] tested the swelling behavior of synthetic hydrogels (polyacrylamide and acylate-based) hydrogels, there are currently no studies that examine these questions for bio-based hydrogels. It is possible that differences in the mechanical strength between bio-based and synthetic hydrogels could influence the pressure the hydrogel can withstand in soil. According to Ahmed [21], synthetic hydrogels possess a higher mechanical strength than bio-based hydrogels, which could be advantageous in withstanding pressure. The challenge thus lies in synthesizing hydrogels with optimized mechanical strength with improved elasticity, that allows the hydrogel to swell.

## 4. Future Research Needs

Here are some of the outstanding questions that need to be addressed regarding the application of bio-based hydrogels as a soil amendment.

1. Additional studies are needed to understand how the particle size distribution of bio-based hydrogels affects soil hydraulic properties. From this review, only one study, Abdallah [57], tested the impact of particle size of a synthetic polyacrylamide hydrogel on soil water retention properties. However, to better understand how new bio-based hydrogels could be tuned to improve certain soil properties, it is important to quantify the specific particle size ranges of bio-based hydrogels. Investigators can then start to determine the relationship between particle size and the hydrogel’s ability to swell in different soils, which has been shown to affect several hydraulic properties like soil water retention and hydraulic conductivity.
2. The particle density of hydrogels can affect soil physical properties, such as porosity and bulk density, which in turn affects how water moves through soil. Studies that investigate how the particle density of various hydrogels affect soil physical properties will help in the development of hydrogels with specific properties that improve soil hydraulic properties. In addition, when comparing the swelling characteristics of various hydrogels at different concentration, it is essential that the hydrogels have similar particle densities. A hydrogel with a high density will have a different swelling characteristic, compared to one with a low density. Therefore, to make accurate comparisons between hydrogels, the particle densities should be determined.
3. Most studies tend to test the effects of hydrogel on sandy soils. Though the impacts of hydrogel application to finer soils, such as clay and silt, are currently not definitive, there is value in investigating the impact of hydrogels over a large range of soil textures.
4. Most studies in literature currently apply hydrogel in powdered or granular form by mixing with soil. More research into different application methods to ascertain the effectiveness of those methods, e.g., spraying in liquid form, applying hydrogels in swollen form, or applying hydrogels in dry solid form, is needed. Some investigators suggest that the hydrogels should be applied after they have been swelled. Studies are needed to quantify the benefit of applying swollen hydrogels and, if useful, to determine how to effectively apply swollen hydrogels.
5. There are limited studies on the impacts of hydrogels on soil unsaturated hydraulic conductivity (*K*). Most studies concentrate on the effects of hydrogels on saturated hydraulic conductivity and in laboratory experiments, likely due to the ease of measuring *Ks* compared to *K*. However, under field conditions, soils will mostly be unsaturated, thus more research is needed to understand how hydrogels affect K.
6. When hydrogels are applied to soil, the surrounding soil tends to exert a force against the hydrogel, hence reducing the hydrogel’s swelling capacity. Research is needed to design hydrogels that can withstand the various biotic, abiotic, and mechanical stresses that soil exerts on hydrogels over at least one growing season.
7. Once a complete data set is established, a predictive mathematical model can be developed to summarize our understanding of the effects of the hydrogels on soil hydraulic properties. For example, can we predict the concentration of hydrogels that when applied to a specific soil decreases/increases *Ks*? This information will increase the usefulness of this knowledge so, for example, farmers know the amount of hydrogel to apply when a particular soil is used to grow a crop. Secondly, if that range of suitable hydrogel application is obtained, is it system dependent e.g., hydrogel type, soil type, climate, soil temperature, or can that recommended range be generalized to all hydrogels and soil types?

## 5. Summary and Conclusions

This review elucidates the impacts of various synthetic and bio-based hydrogels on soil hydraulic properties. This review finds that:

1. Both synthetic and bio-based hydrogels were effective at increasing soil water retention when applied within a range of 0.1 to 1% hydrogel (w/w). Though the increase in water retention was definitive in sandy soils, few studies tested other soil textures.
2. The impact of hydrogels on saturated hydraulic conductivity (*Ks*) was found to be the most inconsistent. Studies on the effect of bio-based hydrogels on *Ks* were fewer than for synthetic hydrogels. Bio-based hydrogels were found to decrease *Ks* by up to 60% in sandy soils. The overwhelming evidence for a decrease in *Ks* was with synthetic hydrogels. The high swelling capacity of synthetic hydrogels stores more water initially when water starts infiltrating soil, reducing the amount of water percolating into the deeper layers and hence decreasing *Ks*. Unsaturated hydraulic conductivity (*K*) was found to decrease at lower matric suctions and increased at higher matric suctions. However, few studies exist that investigate the impact of hydrogels on *K*.
3. The application of synthetic hydrogels mostly reduced soil water infiltration by up to 90%. Only one study was found to measure the impacts of bio-based hydrogel on soil water infiltration, which also confirmed a decrease in infiltration. Hydrogels alter soil structure decreasing the number of drainage pores and retaining water.
4. Like soil water infiltration, hydrogel application mostly decreased soil evaporation as soil water is bound to the hydrogel, reducing how much water is lost to the atmosphere. Hydrogels near the soil surface can also increase evaporation by storing water making it easy for stage one of evaporation to occur.
5. In conclusion, the performance of both synthetic and bio-based hydrogels on soil hydraulic properties will depend on the type of hydrogel, soil texture, application rate, particle size distribution of the hydrogel, the swelling capacity of the hydrogel, the location of placement, and how these properties vary over time.

## Figures and Tables

**Table 1:** Summary of the impacts of synthetic and bio-based hydrogels on soil hydraulic properties.

Reference	Type of Study	Soil Textures	Hydrogel Application Rate	Hydrogel Used	Water Retention	Ks	Soil Water Infiltration	Evaporation
1. Abdallah [57]	Lab and greenhouse study	Sandy soil	0.3 and 0% (w/w)	WaterSorb (WS) (Synthetic)	Gravimetric water content increased by 260%	Decreased 38.9–68.8%	N/A	N/A
2. Abedi-Koupai et al. [55]	Lab	Sandy loam, Loamy, and Clay	2, 4, 6, and 8 g hydrogel/kg soil	PR3005A and Tarawat A100 (Synthetic)	Available water content increased 180% in clay and 220–320% in loamy and sandy loam	N/A	N/A	N/A
3. Agaba et al. [79]	Greenhouse	Sand, Sandy loam, Loam, Silt loam and Clay	0, 0.2, and 0.4% (w/w)	Luquasorb hydrogel, a powder type of potassium polyacrylate (Synthetic)	Plant available water increased 300% in sand, 200% in silt loam	N/A	N/A	N/A
4. Agaba et al. [80]	Greenhouse	Sandy soil	0, 0.2, and 0.4% (w/w)	Luquasorb hydrogel, a powder type of potassium polyacrylate (Synthetic)	100% increase in retained water in top 25 cm of soil	N/A	N/A	N/A
5. Akhter et al. [69]	Potted study in lab	Sandy loam, and Loam	0.1, 0.2 and 0.3% (w/w)	Acrylamide-based hydrogel (Synthetic)	Increased soil water content at field capacity by 17–46% in sandy loam and 23–50% in loam	N/A	N/A	N/A
6. Bai et al. [70]	Lab and potted study	Sandy clay loam	0, 0.05, 0.1, 0.2 and 0.3% (w/w)	Polyacrylate/polyacrylamide-based hydrogels (Synthetic)	Relative soil moisture increased 6.2–32.8%	N/A	N/A	N/A
7. Cannazza et al. [50]	Greenhouse potted study	Red soil (Clay soil) and white soil	0, 0.2, 0.5, 1.0 and 1.5% (w/w)	Cellulose-based hydrogel (Bio-based)	Increased water retention by 50%	N/A	N/A	N/A
8. Koupai et al. [109]	Lab and field study	Sandy loam and Clay	4 and 6 g/kg soil	Superab A200 (Synthetic)	Available water content increased by 230%	N/A	N/A	N/A
9. Leciejewski [81]	Lab study	Loamy sand	0.02, 0.08, 0.17, and 0.25% (w/w)	Potassium polyacrylate-based hydrogel (Synthetic)	Soil water increased by 200–250%	N/A	N/A	N/A
10. Liao et al. [71]	Potted in lab	Sandy loam	0, 0.01, 0.03, 0.06%	Polyacrylamide and acrylic acid-based hydrogel (Synthetic)	Soil water content increased by 2.7–26.5%	N/A	N/A	N/A
11. Montesano et al. [59]	Lab study	Sandy soil	0, 0.5, 1 and 2% (w/w)	Cellulose-based hydrogel (Bio-based)	Increased soil water content at FC by 400%	N/A	N/A	N/A
12. Sarmah & Karak [110]	Lab study	Silty and Sandy	0.1 and 0.25%	Starch based hydrogel (Bio-based)	Water holding capacity increased by 120%	N/A	N/A	N/A
13. Saha et al. [72]	Lab study	Fine sand, Silt loam and Clay	0, 0.1, 0.2, and 0.4% (w/w)	Stockosorb, acrylic-based polymer with acrylamide cross-linking. (Synthetic)	Plant available water capacity increased by 120–330% in fine sand	N/A	N/A	N/A
14. Abrisham et al. [73]	Field study	Sandy loam	0, 1, and 3 g hydrogel/dm^−3^ of soil	Stockosorb, an acrylamide/acrylic acid copolymer potassium Salt. (Synthetic)	Available water content increased by 21.5%	N/A	Soil water infiltration decreased by 21.5%	N/A
15. Bhardwaj et al. [60]	Lab study	Sandy soil	0, 0.5, 2.5, and 5.0 g hydrogel/kg of soil	Cross-linked acrylamide or acrylic acid copolymers (Synthetic)	Increased	Decreased then an increase with time	N/A	N/A
16. Andry et al. [28]	Lab study	Sandy soil	0, 0.1, and 0.2% (w/w)	Carboxymethylcellulose (bio-based) and isopropyl acrylamide (Synthetic)	Available water content increased by 400–500%	Increased	N/A	N/A
17. Lentz [78]	Potted study and lab study	Degraded calcareous Silt loam	0.25 or 0.5% dry weight (5.6 or 11.2 Mg ha^−1^)	Polyacrylamide copolymer and polyacrylic acid-potassium salt hydrogels. (Synthetic)	Plant available water increased by 42%	N/A	N/A	N/A
18. Shahid et al. [74]	Lab study	Sandy loam soil	0.1, 0.2, 0.3 and 0.4% (w/w)	Poly (Acrylamide-co-acrylic acid)/AlZnFe_2_O_4_ nanocomposite hydrogels (Synthetic)	Water retention at field capacity increased by 60–100%	Decreased by 16–63%.	N/A	N/A
19. Hayat & Ali [111]	Lab and potted greenhouse study	Sandy loam	0, 0.25, 0.5, 0.75, 1.00, 1.25, and 1.50% (w/w)	Aquasorb (Synthetic)	Soil moisture content increased by 30–850%	N/A	N/A	N/A
20. Yu et al. [104]	Lab	Loamy sand, Sandy Loam, Sandy clay loam and Clay loam	0.5% (w/w)	WOTE, GNKH, PR3005S, and BJ-210 lXM (Synthetic)	Water absorption capacity increased by two orders of magnitude	N/A	N/A	Decreased evaporation up to 338% after 7 h of drying
21. Banedjschafie & Durner [58]	Lab	Sand	0, 0.3, 0.6, and 1% w/w	Superab A200 (Synthetic)	Plant available water increased by 18%	N/A	N/A	N/A
22. Baran et al. [112]	Lab	Loamy sand and Sand	0, 0.2, 0.6, 1%, and 2% (w/w)	AgroaquaGel (Synthetic)	Increased maximum water capacity by 32–69%	N/A	N/A	N/A
23. Demitri et al. [63]	Lab and greenhouse	Red soil	0.2, 0.5, and 1% (w/w)	Cellulose-based hydrogel (Bio-based)	Increased	N/A	N/A	N/A
24. Geesing [82]	Lab	Loam, Silty clay loam and Sandy loam	0, 1, 3, or 5 g/L of soil	Sodium polyacrylate (Synthetic)	Increased only at rate > 3 g/L	N/A	N/A	N/A
25. Hu et al. [66]	Lab	Sandy loam	0, 2 and 4 (t/ha)	Biomaterials and polyacrylamide (Synthetic and bio-based)	Soil water content increased by 12.1–23.4%	Increased 91–122%.	N/A	N/A
26. Dehkordi [62]	Greenhouse	Sandy soil	0,0.20, 0.40 and 0.6% (w/w)	Superab A200 (Synthetic)	Soil water retention increased 175–375%	N/A	N/A	Evaporation rate decreased by 80% on the third day
27. Narjary et al. [64]	Lab	Sand, alluvial Sandy loam, red Sandy loam and black Clay	0, 0.7, and 0.5% (w/w)	Pusa, a polyacrylate cellulose-based hydrogel. (Bio-based)	Soil water content increased by 400% in sandy soil at soil pressures of 10–100 kPa.	Decreased by 118, 708, and 95% in sand, red sandy loam and alluvial sandy soil, respectively	N/A	N/A
28. Narjary & Aggarwal [65]	Field*	Sandy loam	0, 2.5, and 5 (kg/ha)	Pusa, a polyacrylate cellulose-based hydrogel. (Bio-based)	Plant available water capacity increased by 6–8%	Decreased 45–60%	N/A	N/A
29. Salim [113]	Lab and field	Sandy loam	0, 4, 8, and 12% (w/w)	Sky Gel, copolymer of acrylic acid and sodium acrylic acid (Synthetic)	Water holding capacity increased by 63.2–302.8%	N/A	N/A	N/A
30. Śpitalniak et al. [114]	Lab	Coarse sand, Loamy sand, and Sandy loam		Water absorbent geocomposite (Synthetic)	Soil water retention increased by 54.8–191.6%	N/A	N/A	N/A
31. Zhao et al. [77]	Lab	Sandy loam	0,0.1, 0.2, 0.5, and 1% (w/w)	Acrylamide -based hydrogel (Synthetic)	Soil water content increased by 0.76–3.74%	N/A	Mean infiltration rate decreased by 9–51.5%	N/A
32. Alkhasha et al. [45]	Lab	Loamy sand	0, 0.2, 0.4, 0.6, and 0.8% (w/w)	PagriSap (polyacrylamide-based hydrogel) (Synthetic)	Soil moisture increased by 2.49–5.53%	Decreased 31.4–71.4%	Cumulative infiltration increased from 9.32–21.87%	The 0.2% treatment decreased cumulative evaporation by 10.77% while 0.4–0.8% decreased cumulative evaporation by 6.87–14.86%
33. Alkhasha & Al-Omran [75]	Lab	Sandy loam	0, 0.2, 0.4, 0.6, and 0.8% (w/w)	PagriSap (polyacrylamide- based hydrogel) (Synthetic)	Soil water content increased by 3.3%	N/A	N/A	N/A
34. Al-Humaid & Moftah [76]	Field	Sandy soil	0.1%, 0.2%, 0.4% or 0.6% (w/w)	Stockosorb K400, a cross-linked polyacrylamide (Synthetic)	Soil water content increased by 13.3–300%	N/A	N/A	N/A
35. Zhuang et al. [83]	Lab	Sandy soil	0, 0.08, 0.2, 0.5 and 1%	Sodium polyacrylate (Synthetic)	Maximum water supply quantity increased by 45.61–318.89%	Decreased by 42.53–96.5%.	Decreased	N/A
36. Song et al. [10]	Lab	Sandy loam soil	0, 0.375, 0.650, 0.975% (w/w)	Lignin-based sodium alginate hydrogel (Bio-based)	Maximum water holding capacity in soil increased by 2.98–8.96%	Decreased 63.2–89.5%	N/A	N/A
37. Passauer et al. [38]	Lab	Coarse silica	0, 0.1, 0.25, and 0.5% (w/w)	Lignin-based hydrogel (Bio-based)	Soil water content increased by 300–400%	N/A	N/A	N/A
38. Kashkuli & Zohrabi [115]	Lab	Sandy soil	0, 0.03, 0.06, 0.08, 0.2, and 0.4% (w/w)	Super AB A200 and Herbasorb (Synthetic)	Soil available water increased 350 and 320%	N/A	N/A	N/A
39. Sivapalan [116]	Lab	Sandy	0, 0.03 and 0.07% (w/w)	ALCOSORB^®^ 400 (anionic acrylic copolymer) (Synthetic)	Soil water retention increased 23 and 95%	N/A	N/A	N/A
40. Han et al. [89]	Lab	Sandy loam	ASC or PAM in soil at a mass ratio of 1:2000 (SAP:soil)	Acrylate Sodium Co-polymers (ASC) and Polyacrylamides (PAM) (Synthetic)	N/A	Decrease then an increase with time	N/A	N/A
41. Hussein et al. [88]	Lab	Sandy and Sandy clay loam	0.5, 1.0 and 2.0% (wt/wt)	Poly (acrylic acid)-co-acrylamide hydrogel (Synthetic)	N/A	Decreased by 53.68–87.18% with lower rates (0.5 and 1%) and an increase by 107.6–516.3% at 2%	N/A	N/A
42. Smagin et al. [87]	Lab	Silty sand	0.01 to 0.3 % (w/w)	Technical polyacrylamide (PAA) hydrogel and a co-polymer of acrylamide and (sodium acrylate (Synthetic)	N/A	Decreased by 200–800%	N/A	N/A
43. Mohawesh & Durner [32]	Lab	Sandy soil	0.1, 0.25, and 0.5% (w/w)	Luquasorb (Synthetic)	Soil water content increased up to 86.9%	Decreased by 300%	N/A	N/A
44. Guo et al. [98]	Lab	Sandy loam	0, 0.05, 0.10, 0.15, and 0.20% (w/w)	Poly-γ-glutamic acid-based hydrogel (Bio-based)	Soil water content at FC increased by 8.7–58.3%	N/A	Cumulative infiltration decreased 32.4–52.0%	Cumulative evaporation increased 17.1–25.3%
45. Lentz [99]	Lab	Silt loam, Loam, Loamy sand, and Clay loam	0, 0.25, and 0.5% (w/w)	Polyacrylamide hydrogel (Synthetic)	N/A	N/A	Decreased infiltration by 84–97%	N/A
46. Reddy et al. [100]	Lab	Sandy loam	0, 0.25, 0.75, 1.25 and 1.75%	RDW-W, RDW-I, RDW-W and RDW-F (Synthetic)	N/A	N/A	Maximum reduction of 90% in steady state infiltration	N/A
47. Yang et al. [105]	Lab	Sand, loam, Silt	0.6% (w/w)	Acrylic sodium copolymer (Synthetic)	N/A	N/A	Decrease and increase	Decreased
48. Zhao et al. [106]	Lab	Sandy loam	0, 0.2, 0.5 and 1% (w/w)	Polyacrylamide and acrylic acid-based hydrogel (Synthetic)	N/A	N/A	N/A	Decreased by 0.3–14% at 20 cm on day 30
49. Taban et al. [107]	Lab	Loam and Loamy sand	0.14 and 0.7% (w/w)	Aquasorb PR3005A, a salt copolymer polyacrylamide (Synthetic)	N/A	N/A	N/A	Decreased about 31.25% after 2500 h

## Data Availability

Not applicable.
